# A Maternally Inherited Rare Case with Chromoanagenesis-Related Complex Chromosomal Rearrangements and De Novo Microdeletions

**DOI:** 10.3390/diagnostics12081900

**Published:** 2022-08-05

**Authors:** Jui-Hung Yen, Shao-Yin Chu, Yann-Jang Chen, Yi-Chieh Su, Chun-Ching Chien, Chun-Ying Weng, Pei-Yi Chen

**Affiliations:** 1 Department of Molecular Biology and Human Genetics, Tzu Chi University, Hualien 97004, Taiwan; 2 Genetic Counseling Center, Hualien Tzu Chi Hospital, Buddhist Tzu Chi Medical Foundation, Hualien 97004, Taiwan; 3 Department of Pediatrics, Hualien Tzu Chi Hospital, Buddhist Tzu Chi Medical Foundation, Hualien 97004, Taiwan; 4 Institute of Clinical Medicine, National Yang Ming Chiao Tung University, Taipei 11221, Taiwan; 5 Department of Life Sciences and Institute of Genome Sciences, National Yang Ming Chiao Tung University, Taipei 11221, Taiwan; 6 Department of Pediatrics, Taipei Veterans General Hospital, Taipei 11217, Taiwan; 7 Department of Education and Research, Taipei City Hospital, Taipei 10341, Taiwan; 8 Laboratory of Medical Genetics, Genetic Counseling Center, Hualien Tzu Chi Hospital, Buddhist Tzu Chi Medical Foundation, Hualien 97004, Taiwan

**Keywords:** complex chromosomal rearrangement (CCR), chromoanagenesis, chromoplex, developmental delay, chromosome microarray analysis (CMA), cytogenetics

## Abstract

Chromoanagenesis is a phenomenon of highly complex rearrangements involving the massive genomic shattering and reconstitution of chromosomes that has had a great impact on cancer biology and congenital anomalies. Complex chromosomal rearrangements (CCRs) are structural alterations involving three or more chromosomal breakpoints between at least two chromosomes. Here, we present a 3-year-old boy exhibiting multiple congenital malformations and developmental delay. The cytogenetic analysis found a highly complex CCR inherited from the mother involving four chromosomes and five breakpoints due to forming four derivative chromosomes (2, 3, 6 and 11). FISH analysis identified an ultrarare derivative chromosome 11 containing three parts that connected the 11q telomere to partial 6q and 3q fragments. We postulate that this derivative chromosome 11 is associated with chromoanagenesis-like phenomena by which DNA repair can result in a cooccurrence of inter-chromosomal translocations. Additionally, chromosome microarray studies revealed that the child has one subtle maternal-inherited deletion at 6p12.1 and two de novo deletions at 6q14.1 and 6q16.1~6q16.3. Here, we present a familial CCR case with rare rearranged chromosomal structures and the use of multiple molecular techniques to delineate these genomic alterations. We suggest that chromoanagenesis may be a possible mechanism involved in the repair and reconstitution of these rearrangements with evidence for increasing genomic imbalances such as additional deletions in this case.

## 1. Introduction

Chromoanagenesis is a novel class of chromosomal rearrangements characterized by massive and highly complex chromosomal changes occurring at one-step cellular events by which genomic alterations such as deletion and duplication can accumulate in an all-at-once manner [1,2,3]. Over the last decade, chromoanagenesis has been described first in cancer cells and in patients with congenital anomalies [4,5,6]. Based on different proposed mechanisms and chromosomal structural complexity, chromoanagenesis consists of three subtypes: chromothripsis, chromoanasynthesis, and chromoplexy [7,8]. Chromothripsis and chromoanasynthesis occur within one or more chromosomes with shattering and reshuffling chromosome segments and undergo different DNA repair mechanisms, such as nonhomologous end-joining (NHEJ) in chromothripsis and fork-stalling and template switching (FoSTeS) in chromoanasynthesis [1]. In contrast, chromoplexy usually generates structural rearrangements across multiple chromosomes involving inter-chromosomal translocation and deletion [9]. Although chromoanagenesis is commonly detected in various human cancers, to date, there are data from only a few germline cases [10,11,12]. These germline chromoanagenesis have been observed in CCRs but also in simple deletion or duplication cases [13,14]. These reports suggest an underestimated situation for these complex events because they would have gone largely undetected by conventional cytogenetics. Molecular techniques such as fluorescence in situ hybridization (FISH), chromosomal microarray analysis (CMA), and next-generation sequencing (NGS) increase the opportunity to uncover these chromosomal rearrangements and allow for further exploration of their mechanism and significance in congenital disease.

This report describes the clinical and genetic analysis of a 3-year-old boy exhibiting developmental delay and facial dysmorphism. CCRs involving four chromosomes (2, 3, 6, and 11) were detected and transmitted from the carrier mother who had mild mental retardation. Using G-banding and FISH analysis, we identified a very rare derivative chromosome 11 that likely results from chromosomal shattering and subsequent random reassembly of DNA fragments from chromosome 6q and 3q to the q arm terminal of chromosome 11, which are characteristics of constitutional chromoplexy [7,9]. CMA studies further revealed one submicroscopic deletion at 6p12.1 (627 kb) inherited from the mother and two additional de novo deletions at 6q14.1 (915 kb) and 6q16.1~6q16.3 (2.1 Mb). This study highlights the importance of the detailed delineation of complex rearrangements and the impact of congenital chromoanagenesis.

## 2. Materials and Methods

### 2.1. Patient and Clinical Examination

A 3-year-old boy with psychomotor retardation and language delay was referred to genetic counseling at Tzu-Chi General Hospital, Hualien, Taiwan. His mother, with mild mental retardation, was 35 at the time of delivery. The child was born by cesarean section with a birth weight of 2959 gm, length of 49 cm and head circumference of 34 cm. General cyanosis and bradycardia after birth were observed. At that time, he was admitted to the ICU for 26 days and treated under the diagnosis of meconium aspiration syndrome. A cephalohematoma over the left parietal area was noted, but the pediatric brain echo revealed normal ventricles and normal choroid plexus pulsation.

At one year of age, brain magnetic resonance imaging (MRI) showed corpus callosum dysgenesis and a mild delay in myelination. Speech delay and psychomotor retardation were described. The body weight of 6.6 kg, height of 67 cm and head circumference of 42.3 cm all fall below the third percentile on the growth curve in the normal population.

**Figure 1:**
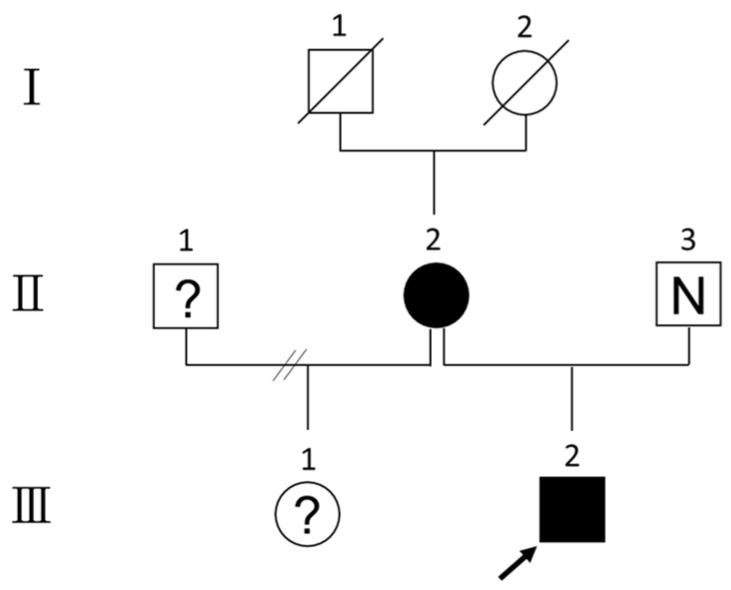
Pedigree of the family. Squares or circles with “?” denote individuals whose genotype information is not available. Dark squares or circles denote affected individuals. Square with “N” denotes the individual with a normal karyotype. The grandfather (I-1) and grandmother (I-2) of the child are deceased. The mother (II-2) with her previous husband (II-1) has one daughter (III-1), but they are not available for clinical or genetic testing. The arrow represents the child (III-2), who harbors the same complex chromosomal alterations as his mother. The father (II-3) has a normal karyotype.

At age 3, his facial dysmorphism was observed, including microcephaly, bilateral temporal narrowing, prominent nasolabial fold, relatively pale appearance and abnormal head shape. Hypotelorism, short stature, speech delay and psychomotor retardation persisted at that time. The mother had one daughter in her previous marriage (Figure 1), and this daughter was not available for clinical or genetic testing. The proband was the only child in the mother’s second marriage. All family members gave their written consent after all the details of the study were fully explained.

### 2.2. Karyotyping and FISH Analysis of Cultured Blood Lymphocytes

Conventional cytogenetic analysis was performed using the standard GTW-banding method at 550 bands of resolution on cultured blood lymphocytes [15]. The chromosomal rearrangements were further evaluated using FISH on the metaphase chromosome spreads based on the manufacturer’s protocol. Briefly, spectral karyotyping (SKY) using 24-color-labeled painting probes (Applied Spectral Imaging, Carlsbad, CA, USA) was performed to improve identification of inter-chromosomal rearrangements. FISH was used to further determine the CCRs structure and the subtelomeric region of the der(11) using five specific probes, including CEP11-FITC, WCP3-Texas Red, WCP6-FITC, 6qter-FITC and 11qter-Texas Red (Cytocell, Inc., Adderbury, Oxfordshire, UK). The 6qter-FITC (D6S2522) and 11qter-Texas Red (D11S4974) are sub-telomeric specific probes and contain unique DNA located close to 6q and 11q telomeres, respectively.

### 2.3. Chromosome Microarray Analysis of Peripheral Blood

CMA was performed using CytoOneArray^®^ (Phalanx Biotech, Hsinchu, Taiwan), which contained 33,255 probes with 10–30 kb resolution for more than 300 disease regions. This platform was designed to analyze copy number variation (CNV), especially in pediatric patients with developmental delays and intellectual disabilities. The experimental procedures followed the protocol provided by the manufacturer. The array data were described based on the reference genome version of GRCh37, following the guidelines of An International System for Human Cytogenomic Nomenclature (ISCN2020). The clinical significance of CNV was analyzed using International Collaboration for Clinical Genomics (ICCG) (https://clinicalgenome.org/) (accessed on 14 June 2022) and Online Mendelian Inheritance in Man (OMIM) (http://www.omim.org/) (accessed on 14 June 2022).

## 3. Results

**Figure 2:**
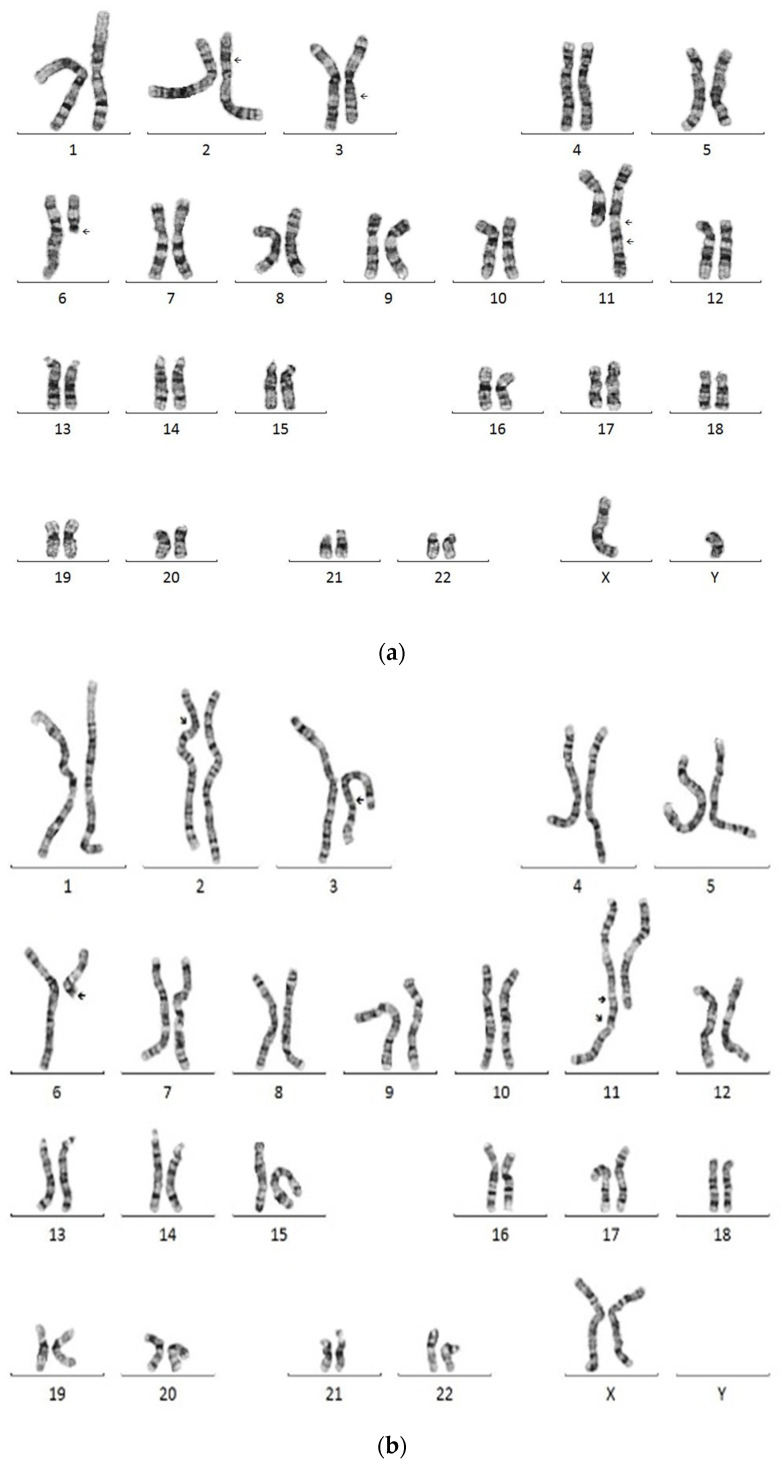
Cytogenetic characterization of the cultured blood lymphocytes. Conventional G-banding analysis of cultured blood lymphocytes showed a complex karyotype: 46,XY,der(2)t(2;6)(p16;q21), der(3)t(2;3)(p16;q21),del(6)(q13),der(11)ins(11;6)(q25;q13q21)t(3;11)(q21;q25) in the child (**a**). A similar complex GTW-banding pattern was seen in the mother (**b**). The der(11) chromosome marked with two breakpoints that probably derived from insertion of 6q13~6q21 into band 11q25, and translocation of the segment 3q25~3qter to chromosome 11 at band 11q25. Cytogenetic nomenclature is based on ISCN 2020. The arrows indicate breakpoints.

The pedigree of the family is shown in Figure 1. Cytogenetic analysis of the child found a CCR result with four-way translocations between the short arm of chromosome 2 and the long arms of chromosomes 3, 6, and 11, as well as an insertion by the fragment of 6q13 to 6q21 into the breakpoint at 11qter (Figure 2a). The proband’s karyotype was reported as 46,XY,der(2)t(2;6)(p16;q21),der(3)t(2;3)(p16;q21),del(6)(q13),der(11)ins(11;6) (q25;q13q21)t(3;11)(q21;q25). Further cytogenetic analysis of his parents showed that the father exhibited a normal 46,XY karyotype, while the mother had an identical result as the proband’s karyotype (Figure 2b), indicating that these chromosomal aberrations are maternally inherited.

**Figure 3:**
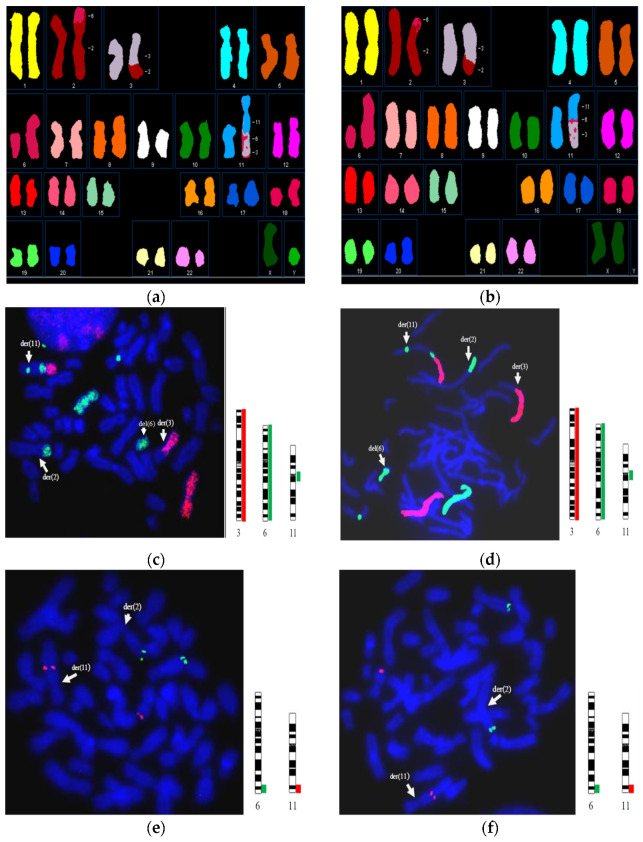
Molecular cytogenetic analysis of the chromosomal rearrangements. SKY using 24-color SKY probes showed similar complex chromosomal changes as G-banding in the child (**a**) and the mother (**b**). Four abnormal chromosomes, der(2), der(3), del(6) and der(11), were detected using WCP3-Texas Red, WCP6-FITC and CEP11-FITC probes by metaphase FISH; and der(11) contained partial fragments from chromosomes 3 and 6 in the child (**c**) and his mother (**d**). Metaphase FISH using 6qter-FITC and 11qter-Texas Red sub-telomeric probes showed that the 11q telomere was retained in the der(11) chromosome in the child (**e**) and the mother (**f**).

Similar findings were detected in FISH analysis (Figures 3). The der(11) connecting the long arm of chromosome 11 with the fragment from chromosome 6 to another fragment from chromosome 3 was detected in the proband and his mother (Figure 3c,d). In addition, a 11qter-Texas Red (D11S4974) sub-telomere probe was used to elucidate whether the terminal region of chromosome 11 was retained or translocated to other chromosomes. Our results showed that the terminal segment of chromosome 11q was retained and located in the near-middle region of derivative chromosome 11 (Figure 3e,f).

**Figure 4:**
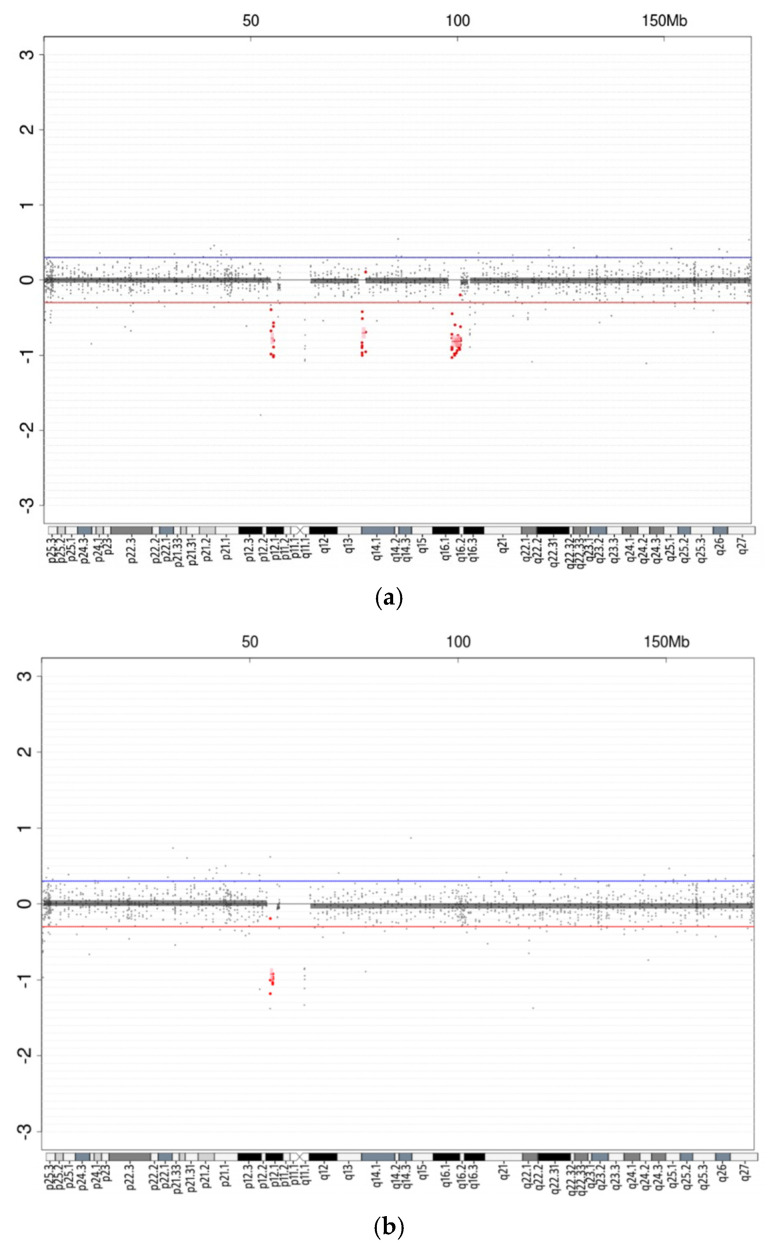
Chromosomal microarray analysis (CMA) using blood lymphocytes. Microarray studies revealed three deleted loci on chromosome 6, including one inherited deletion in 6p12.1 and two deletions in 6q14.1 and 6q16.1~q16.3 region in the child (**a**), and one deletion in 6p12.1 in the mother (**b**). The “Log_2_ Ratio = 0” represents a normal CNV result; “Log_2_ Ratio < −0.3” represents a deletion (below red line); and “Log_2_ Ratio > 0.3” represents a duplication (above blue line).

To further evaluate any possible genomic imbalance existing in the proband and his mother, we examined CNV using a chromosomal microarray. The CMA report of the mother was arr[GRCh37] 6p12.1 (54906732_55533279)x1, and the child was arr[GRCh37] 6p12.1 (54906732_55533279)x1, 6q14.1 (76909657_77824306)x1, 6q16.1q16.3 (98650510_ 100809778)x1. As shown in Figure 4, one microdeletion was detected on the short arm of chromosome 6, within region 6p12.1, spanning approximately 627 kb, inherited from his mother. Analysis of the predicted intolerance for loss of function and associated with haploinsufficiency of these genes using ICCG and OMIM database are listed in Table 1. This microdeletion region encompassed three Online Mendelian Inheritance In Man (OMIM) genes: *HCRTR2* (* 602393), *GFRAL* (* 617837) and *HMGCLL1* (* 619050). Moreover, the child had two de novo microdeletions on the long arm of chromosome 6 within the regions of 6q14.1 and 6q16.1~6q16.3. Deletion of 6q14.1 spanned 915 kb and contained three pseudogenes: *RNU6-261P*, *RNU6-84P* and *LOC100131680*. Deletion of 6q16.1~q16.3 spanned approximately 2.16 Mb and encompassed 20 genes, including 8 OMIM genes: *POU3F2* (* 600494), *FBXL4* (* 605654), *COQ3* (* 605196), *PNISR* (* 616653), *USP45* (* 618439), *CCNC* (* 123838), *PRDM13* (* 616741) and *MCHR2* (* 606111). Our present case improves the understanding of the characteristics of chromoanagenesis in familial CCR.

## 4. Discussion

**Figure 5:**
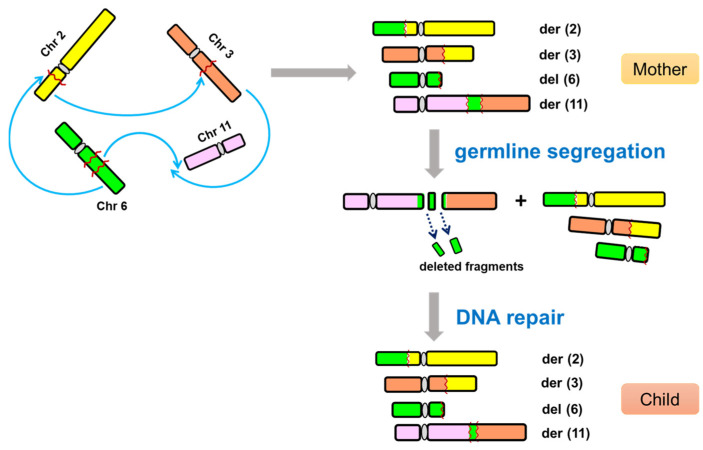
A proposed model of chromoanagenesis-related CCR in this study. This model illustrates a possible mechanism of the familial CCR through germline segregation and DNA repair to generate de novo deletions in the child.

Molecular characterization using multiple cytogenomic methods constitutes a powerful tool for understanding the cryptic abnormalities of complex chromosomal disorders. Here, we report a child with severe global developmental delay in the presence of a CCR karyotype. Using FISH analysis, we further confirmed a derivative chromosome 11 with the pattern of joining of chromosomal fragments from chromosomes 3, 6, and 11, which strongly resembles the new chromosomal rearrangements termed chromoanagenesis. We postulated that a similar mechanism might also drive the formation of novel genetic imbalances in the child because CMA studies revealed one inherited deletion and two additional deletions at chromosome 6 (Figure 5). These 6q deletions suggest a chromoanagenetic phenomenon because the complex derivative chromosome 11 has an insertion fragment from 6q13 to 6q21 that covers those deleted regions. It is possible to generate breaks and reunions and then create novel deletions within the complex der(11) chromosome during the process of germline segregation and DNA repair, which may play a role in the patient’s developmental delay.

The 11q terminal region, band 11q25, was probably disrupted by the insertion of 6q fragment. It is important to delineate the 11q25 band is distal or proximal to the 6q13→q21 region. Our FISH studies (Figure 3e,f) only supported the 11q telomere was retained in the der(11) chromosome. Further studies using 11qter-Texas Red and WCP6-FITC probes may be considered to determine the relative position between band 11q25 and 6q13 to 6q21 region on the der(11) chromosome.

Our studies cannot completely exclude deletions in 6q14.1 and 6q16.1q16.3 are paternal inheritance because lack of data for the father’s microarray. However, the possibility of deletions co-occurring in chromosome 6 at breakpoints 6q13 and 6q16.1 is extremely low. In addition, the father has a normal phenotype, so we think that microdeletions of 6q13 and 6q16.1 are more likely de novo in order to explain the proband’s aggravated phenotype.

Based on the abovementioned structural aberrations in the mother and her child, their chromosomal rearrangements are suspected to be associated with chromoanagenesis [16]. Chromoanagenesis is now regarded as an important driver of cancer evolution and the generation of multiple developmental disorders [2,17,18]. Fukami et al. described that chromoanagenesis events could produce intra-chromosomal deletions, duplications, inversions, and translocations, as well as inter-chromosomal translocations [19]. They also suggested that germline chromoanagenesis of autosomes often results in developmental delay and dysmorphic features, whereas X chromosomal rearrangements are usually associated with relatively mild clinical manifestations. Such observations lead to a deeper study of the genomic aberrations of germline CCR, many of which go undetected by traditional cytogenetic and molecular methods.

In this study, the maternally inherited deleted region encompassed the *HCRTR2*, *GFRAL* and *HMGCLL1* genes. Interestingly, these gene-encoded proteins are all involved in the regulation of metabolism: (1) the *HCRTR2* gene encodes a G-protein coupled receptor for both orexin-A and orexin-B neuropeptides in the regulation of feeding behavior; (2) *GFRAL* encodes a brainstem-restricted receptor for GDF15 that regulates food intake, energy expenditure and body weight; and (3) the *HMGCLL1* gene encodes a 3-hydroxymethyl-3-methylglutaryl-CoA lyase involved in ketone body biosynthesis. The impact of this CNV may account for some clinical symptoms in the affected mother and her child. However, the clinical predicted values of haploinsufficiency for those genes are not very significant (Table 1); thus, the expression levels of these deleted genes may need further confirmation by RT-qPCR. Long-term follow-up and evaluation are needed to clarify the real impact of this region’s absence on this family's clinical symptoms.

The child has a significantly de novo deletion within 6q16.1~q16.3 spanning 2.16 Mb. This CNV was previously reported in patients with developmental delay [20,21]. The following nine genes were included in this region: POU3F2, FBXL4, FAXC, COQ3, PNISR, USP45, TSTD3, CCNC, PRDM13 and MCHR2. Eight genes overlapped with a Japanese case with developmental delay due to a 6q16.1 deletion, except for the *MCHR2* gene [21]. *MCHR2* encodes a G protein-coupled receptor for a melanin-concentrating hormone expressed in the central and peripheral nervous systems and plays an important role in controlling feeding behaviors and energy metabolism [22,23]. Several phenotypic discordances, such as abnormal brain MRI, birth cyanosis and bradycardia, were observed in our patient but not in the Japanese case. This suggests that the *MCHR2* gene may play a role in the severe global developmental delay in this case. The neuronal transcription factor POU3F2 is important for hypothalamic development and function. POU3F2 deletion has been well-described to have a haploinsufficient effect in patients with developmental delay, intellectual disability and impairments in speech and language skills [20,24,25]. Therefore, POU3F2 is considered, at least to some extent, a putative candidate gene associated with the clinical symptoms of the proband. FBXL4 is another important OMIM gene that has been reported and associated with mitochondrial DNA depletion syndrome 13 (encephalomyopathic type). However, FBXL4-related disorder is inherited in an autosomal recessive manner; thus, heterozygous carriers are considered phenotypically unaffected [26,27].

## 5. Conclusions

In conclusion, we have identified aberrations in a chromoanagenesis-related CCR case with two additional 6q deletions. The genomic abnormalities of the patient were characterized by G-banding, FISH, SKY and MCA to improve the understanding of the relationship between genotype and phenotype. We suggest that a 6q deleted region of 2.1 Mb supports the effect of haploinsufficiency on the severe developmental delay in this patient. Possible involvement of MCHR2 in the control of feeding behavior and energy metabolism and POU3F2 in developmental delay, intellectual disability and speech impairment is also hypothesized.

## Figures and Tables

**Table 1:** Dosage sensitivity results.

CMA Deletions	Gene/Region	HI Score	OMIM No.	%HI	pLI	LOEUF
6p12.1 (54906732_55533279)x1	*HCRTR2*	Not Yet Evaluated	602393	38.52	0.01	0.64
	*GFRAL*	Not Yet Evaluated	617837	59.12	0	1.26
	*HMGCLL1*	Not Yet Evaluated	619050	31.99	0	1.07
6q14.1 (76909657_77824306)x1	*RNU6-261P*	−1 (Pseudogene)	-	-	-	-
	*RNU6-84P*	−1 (Pseudogene)	-	-	-	-
	*LOC100131680*	−1 (Pseudogene)	-	-	-	-
6q16.1q16.3 (98650510_100809778)x1	*POU3F2*	Not Yet Evaluated	600494	15.19	0.92	0.38
	*FBXL4*	Not Yet Evaluated	605654	10.28	0	0.92
	*MIR548AI*	Not Yet Evaluated	-	-	-	-
	*BDH2P1*	−1 (Pseudogene)	-	-	-	-
	*FAXC*	Not Yet Evaluated	-	21.43	0.93	0.36
	*COQ3*	Not Yet Evaluated	605196	40.91	0	1.34
	*PNISR*	Not Yet Evaluated	616653	9.32	1	0.18
	*LOC100506090*	−1 (Pseudogene)	-	-	-	-
	*LOC101927365*	−1 (Pseudogene)	-	-	-	-
	*USP45*	Not Yet Evaluated	618439	45.27	0	1.06
	*TSTD3*	Not Yet Evaluated	-	-	-	-
	*CCNC*	Not Yet Evaluated	123838	2.45	1	0.15
	*RPS3P5*	−1 (Pseudogene)	-	-	-	-
	*PRDM13*	Not Yet Evaluated	616741	56.21	0.56	0.46
	*MCHR2*	Not Yet Evaluated	606111	40.65	0	1.2
	*MCHR2-AS1*	Not Yet Evaluated	-	-	-	-
	*NPM1P38*	−1 (Pseudogene)	-	-	-	-
	*LOC100420742*	−1 (Pseudogene)	-	-	-	-
	*PRDX2P4*	−1 (Pseudogene)	-	-	-	-
	*LOC100129854*	−1 (Pseudogene)	-	-	-	-

HI score: Haploinsufficiency score; %HI: DECIPHER Haploinsufficiency index, value less than 10% predict that a gene is more likely to exhibit haploinsufficiency; pLI: gnomAD pLI score, value greater than or equal to 0.9 indicate that a gene appears to be intolerant of loss of function variation; LOEUF: gnomAD predicted loss of function, value less than 0.35 indicate that a gene appears to be intolerant of loss of function variation.

## Data Availability

Not applicable.
